# DNA methylation-mediated differential expression of *DLX4* isoforms has opposing roles in leukemogenesis

**DOI:** 10.1186/s11658-022-00358-0

**Published:** 2022-07-26

**Authors:** Jing-dong Zhou, Yang-jing Zhao, Jia-yan Leng, Yu Gu, Zi-jun Xu, Ji-chun Ma, Xiang-mei Wen, Jiang Lin, Ting-juan Zhang, Jun Qian

**Affiliations:** 1 Department of Hematology, Affiliated People’s Hospital of Jiangsu University, 8 Dianli Rd., Zhenjiang, 212002 Jiangsu People’s Republic of China; 2 Zhenjiang Clinical Research Center of Hematology, Zhenjiang, 212002 Jiangsu People’s Republic of China; 3 The Key Lab of Precision Diagnosis and Treatment in Hematologic Malignancies of Zhenjiang City, Zhenjiang, 212002 Jiangsu People’s Republic of China; 4 Jiangsu Key Laboratory of Medical Science and Laboratory Medicine, School of Medicine, Jiangsu University, Zhenjiang, 212013 Jiangsu People’s Republic of China; 5 Laboratory Center, Affiliated People’s Hospital of Jiangsu University, 8 Dianli Rd., Zhenjiang, 212002 Jiangsu People’s Republic of China; 6 Department of Oncology, Affiliated People’s Hospital of Jiangsu University, 8 Dianli Rd., Zhenjiang, 212002 Jiangsu People’s Republic of China

**Keywords:** *DLX4*, Expression, Methylation, Function, Leukemogenesis

## Abstract

**Background:**

Previously, we reported the expression of *DLX4* isoforms (*BP1* and *DLX7*) in myeloid leukemia, but the functional role of *DLX4* isoforms remains poorly understood. In the work described herein, we further determined the underlying role of *DLX4* isoforms in chronic myeloid leukemia (CML) leukemogenesis.

**Methods:**

The expression and methylation of *DLX4* isoforms were detected by real-time quantitative PCR (RT-qPCR) and real-time quantitative methylation-specific PCR (RT-qMSP) in patients with CML. The functional role of *DLX4* isoforms was determined in vitro and in vivo. The molecular mechanism of *DLX4* isoforms in leukemogenesis was identified based on chromatin immunoprecipitation with high-throughput sequencing (ChIP-Seq)/assay for transposase-accessible chromatin with high-throughput sequencing (ATAC-Seq) and RNA sequencing (RNA-Seq).

**Results:**

*BP1* expression was increased in patients with CML with unmethylated promoter, but *DLX7* expression was decreased with hypermethylated promoter. Functionally, overexpression of *BP1* increased the proliferation rate of K562 cells with S/G2 promotion, whereas *DLX7* overexpression reduced the proliferation rate of K562 cells with G1 arrest. Moreover, K562 cells with *BP1* overexpression increased the tumorigenicity in NCG mice, whereas K562 cells with *DLX7* overexpression decreased the tumorigenicity. Mechanistically, a total of 91 genes including 79 messenger RNAs (mRNAs) and 12 long noncoding RNAs (lncRNAs) were discovered by ChIP-Seq and RNA-Seq as direct downstream targets of *BP1*. Among the downstream genes, knockdown of *RREB1* and *SGMS1-AS1* partially revived the proliferation caused by *BP1* overexpression in K562 cells. Similarly, using ATAC-Seq and RNA-Seq, a total of 282 genes including 151 mRNA and 131 lncRNAs were identified as direct downstream targets of *DLX7*. Knockdown of downstream genes *PTPRB* and *NEAT1* partially revived the proliferation caused by *DLX7* overexpression in K562 cells. Finally, we also identified and validated a *SGMS1-AS1*/*miR-181d-5p*/*SRPK2* competing endogenous RNA (ceRNA) network caused by *BP1* overexpression in K562 cells.

**Conclusions:**

The current findings reveal that DNA methylation-mediated differential expression of *DLX4* isoforms *BP1* and *DLX7* plays opposite functions in leukemogenesis. *BP1* plays an oncogenic role in leukemia development, whereas *DLX7* acts as a tumor suppressor gene. These results suggest *DLX4* as a therapeutic target for antileukemia therapy.

**Supplementary Information:**

The online version contains supplementary material available at 10.1186/s11658-022-00358-0.

## Background

Hematopoiesis is a process that is strictly controlled by gene expression regulation that drives differentiation from hematopoietic stem/progenitor cells to mature hematopoietic cells [1]. During leukemogenesis, biological processes including cell proliferation, expansion, self-renewal, and differentiation are disrupted, resulting in an accumulation of immature blast cells [2]. Hematological myeloid malignancies are a group of disorders characterized by clonal expansion of hematopoietic stem/progenitor cells, including acute myeloid leukemia (AML), chronic myeloid leukemia (CML), myelodysplastic syndromes (MDS) and myeloproliferative neoplasms (MPN). Epigenetic processes such as DNA methylation and histone modifications with roles in regulating gene(s) expression have been proved to play a vital role in myeloid malignancies [3]. Recurrent somatic mutations have been identified in genes involved in epigenetic regulators such as *TET2*, *DNMT3A*, *IDH1*/*2* and *EZH2* that were required for malignant transformation [4, 5]. Furthermore, aberrant DNA hypermethylation of tumor suppressor genes is a frequent event and contributes to luekemogenesis in myeloid malignancies, and could also predict disease progression and clinical outcome [6]. Importantly, demethylation agents including azacytidine and decitabine have been approved as first-line therapy in AML and MDS [7].

The human *DLX4* gene is a member of the *DLX* subfamily of homeobox genes, which has three RNA splicing isoforms encoding different proteins, namely *DLX4* isoform 1 (NM_138281, encoding *BP1*), *DLX4* isoform 2 (NM_001934, encoding *DLX7*), and *DLX4* isoform 3 (encoding an unconfirmed protein). Most previous research on this topic has mainly focused on *DLX4* isoform 1, thus *BP1* is also called *DLX4* in some studies. It has been demonstrated that *DLX4* regulates diverse developmental processes such as skeletal patterning, neurogenesis, and hematopoiesis [8]. Moreover, *BP1* is also implicated in biological progresses and early development, which are frequently dysregulated in human cancers, possible owing to DNA amplification [9, 10]. *BP1* is absent from most normal tissues but is commonly expressed in diverse human cancers including breast cancers and ovarian cancer, lung cancer, and acute leukemia [11–17]. Moreover, high *BP1* expression promotes tumor progression and predicts clinical outcome in various human cancers [15–18]. These results suggest an oncogenic role of *BP1* in human cancers, including leukemia.

Interestingly, epigenetic inactivation of *DLX4* by DNA methylation has been proved in chronic lymphocytic leukemia (CLL) and several other solid tumors [19–23]. Moreover, *DLX4* hypermethylation, with a role in silencing *DLX4* expression, is associated with disease evolution in uterine cervical low-grade squamous intraepithelial lesions (LSILs) and non-small cell lung cancer (NSCLC) [22, 23]. Importantly, our previous studies also confirmed the *DLX4* hypermethylation pattern in myeloid malignancies [24–26], associated with poor prognosis in MDS and AML [24, 25]. The results support the tumor suppressive role of *DLX4* in human cancers, including myeloid malignancies. Superficially, there seems to be a contradiction regarding the role of *DLX4* in tumorigenesis. Notably, we found that *DLX4* hypermethylation was correlated with reduced *DLX7* expression but not *BP1* expression in AML and CML [25, 26]. At the same time, our previous study also reported that *BP1* expression is upregulated in AML, whereas *DLX7* expression is downregulated [27]. These results suggest that *BP1* and *DLX7* may play opposite roles in luekemogenesis.

In the work described herein, we first evaluated the expression of two *DLX4* isoforms, viz. *BP1* and *DLX7*, in CML patients and investigated the potential correlations with clinical pathological parameters. Moreover, we determined the biological roles of *BP1* and *DLX7* in luekemogenesis by using in vivo and in vitro experiments. Finally, the potential molecular mechanism of *BP1* and *DLX7* in leukemogenesis was further explored. Collectively, the findings of the current study may provide broader insights into the understanding of leukemogenesis and provide potential therapeutic targets for antileukemia therapies.

## Methods

### Patients

The current investigation was approved by the Ethics Committee of the Affiliated People’s Hospital of Jiangsu University (no. K-20190016, date 25/02/2019) in accordance with the Declaration of Helsinki, and included a total of 37 healthy controls and 75 CML patients who were treated at our hospital. Clinical and laboratory characteristics of CML patients are presented in Table 1. Bone marrow (BM) samples were obtained from all participants after written informed consent was signed. BM mononuclear cells were separated through density-gradient centrifugation by using lymphocyte separation medium (Solarbio, Beijing, China).

**Table 1:** Correlations of *BP1* and *DLX7* expression with clinical characteristics and laboratory results of CML patients

Patient characteristic	*BP1* expression			*DLX7* expression		
	High (*n* = 25)	Low (*n* = 50)	*P*	Low (*n* = 39)	High (*n* = 36)	*P*
Sex, male/female	16/9	33/17	1.000	26/13	23/13	0.813
Median age, years (range)	54 (25–75)	53.5 (15–83)	0.698	48 (22–76)	52.5 (15–67)	0.150
Median WBC, × 10^9^/L (range)	65.3 (25.1–298.6)	70.3 (20–412.8)	0.348	73.1 (24.5–413.8)	60.9 (21.7–321.9)	0.627
Median hemoglobin, g/L (range)	97 (70–134)	92 (57–145)	0.908	92 (57–145)	96.5 (66–134)	0.585
Median platelets, × 10^9^/L (range)	277 (16–1175)	367 (40–1004)	0.230	259 (40–1006)	373.5 (16–1175)	0.597
Cytogenetics			0.227			0.975
t(9;22)	16	35		27	24	
t(9;22) with additional alteration	5	3		4	4	
Normal karyotype	2	3		2	3	
No data	2	9		6	5	
Staging			0.602			0.008
CP	20	40		26	34	
AP	1	5		5	1	
BC	4	5		8	1	
Median *BCR*/*ABL* transcript, % (range)	164.1 (13.81–14,464.68)	136.74 (16.87–3211)	0.933	216.47 (16.87–3211.00)	93.285 (13.81–14,464.68)	0.054

WBC, white blood cells; CP, chronic phase; AP, accelerated phase; BC, blast crisis

### RNA isolation, reverse transcription, and RT-qPCR

Total RNA was isolated using TRIzol reagent (Invitrogen, Carlsbad, CA) as reported [28–30]. Reverse transcription was performed by using PrimeScript RT reagent kit (TaKaRa, Tokyo, Japan) prepared for mRNA detection and MiScript reverse transcription kit (Qiagen, Duesseldorf, Germany) prepared for micro RNA (miRNA) detection. Real-time quantitative PCR (RT-qPCR) was conducted to determine mRNA (*BP1*, *DLX7*, and their downstream targets) and miRNA (*miR-181d-5p*) expression by using AceQ qPCR SYBR Green Master Mix (Vazyme, Piscataway, NJ) and miScript SYBR green PCR kit (Qiagen, Duesseldorf, Germany) with the primers shown in Additional file 1: Table S1. Relative mRNA/miRNA expression was computed using the 2^−∆∆CT^ method referred to *ABL1*/*U6* expression.

### DNA isolation, bisulfite modification, and RT-qMSP

Genomic DNA was extracted using Puregene Blood Core Kit B (QIAGEN, Duesseldorf, Germany) and was modified by the EpiTect Bisulfite Kit (QIAGEN, Duesseldorf, Germany) [28–30]. Real-time quantitative methylation-specific PCR (RT-qMSP) was performed to detect *BP1* methylation by using AceQ qPCR SYBR Green Master Mix (Vazyme, Piscataway, NJ) using the primers presented in Additional file 1: Table S1. Relative *BP1* methylation was calculated using the 2^−∆∆CT^ method referred to *ALU* methylation.

### BSP

Bisulfite sequencing PCR (BSP) was further applied to investigate and/or confirm *BP1* methylation by using TaKaRa Taq Hot Start Version (Tokyo, Japan) with the primers presented in Additional file 1: Table S1 [31]. Clone sequencing of PCR products was conducted as per previous literature [24–26]. Six independent clones from each sample were sequenced by Sanger sequencing (BGI, Shanghai, China).

### Cell culture and transfection

Human CML cell-line K562 was cultured in Roswell Park Memorial Institute (RPMI) 1640 medium supplemented with 10% fetal calf serum (ExCell, Shanghai, China) under conditions of 5% CO_2_ humidified atmosphere at 37 °C. The complete coding sequences (CDS) of human *BP1*/*DLX7* were inserted into the BamHI/AgeI of GV569 (Ubi-MCS-3FLAG-CBh-gcGFP-IRES-puromycin) vector (Genechem, Shanghai, China), and packaged with lentivirus, then infected into cells according to the manufacturer’s directions. Small interfering RNA (siRNA) duplex oligonucleotides targeting *RREB1*/*SGMS1-AS1*/*PTPRB*/*NEAT1* mRNA with the sequences summarized in Additional file 1: Table S1 were synthesized by GenePharma (Shanghai, China). Transfections of siRNA were conducted by using X-tremeGENE siRNA transfection reagent (Roche, Basel, Switzerland) based on the provided recommendations.

### Western blot

The experimental procedures for western blotting were described previously [28, 29]. Antibodies utilized in this study included rabbit anti-*BP1* (NB100-481) (Novus Biologicals, Littleton, CO) and mouse anti-glyceraldehyde 3-phosphate dehydrogenase (GAPDH) (BOSTER, Wuhan, China).

### Cell proliferation and cell cycle assays

Cell proliferation and cell cycle assays were carried out by cell counting and flow cytometry, respectively. For cell proliferation analysis, cells (1 × 10^5^ cells/ml) were seeded into a six-well plate with complete medium for culture. After culturing for 0, 1, 2, and 3 days, cells were counted in a counting chamber by using an ordinary microscope (manual counting) for three times. For cell cycle analysis, cells (2 × 10^5^ cells/ml) were seeded into a six-well plate with complete medium for culture. The BD Cycletest Plus DNA reagent kit (BD Pharmingen, San Diego, CA) was used to analyze the cell cycle distribution according to the manufacturer’s protocols, followed by analysis using flow cytometry. Each experiment was repeated three times.

### Xenograft mouse model

All mouse experiments were approved by the Committee on the Ethics of Animal Experiments of Jiangsu University (no. UJS-IACUC-AP-20190305073, date: 2019.03.05), also in compliance with the Basel Declaration. Mice of severe immune-deficient strain NCG (NOD/ShiLtJGpt-Prkdc^em26Cd52^Il2rg^em26Cd22^/Gpt) were obtained from GemPharmatech Co, Ltd (Nanjing, China). A total of 1 × 10^6^ tested cells (K562-NC/K562-BP1/K562-DLX7) were injected into each group of 5-week-old female NCG mice through the tail vein respectively. Body weight and peripheral blood of the mice were determined weekly. Growth of the mice affected by the leukemia cells was monitored (IVIS) at the 4th and 6th week. Mice were euthanized when they developed a bowed back and hind limb paralysis.

### RNA-Seq

The details of RNA-Seq were reported in previous studies [32]. The sequencing data were analyzed with the assistance of Genesky Biotechnologies Inc. (Shanghai, China). Differentially expressed genes were analyzed by using Deseq2 software with *P* < 0.05 and |log_2_(fold change)| > 1.

### ChIP-Seq

Chromatin immunoprecipitation (ChIP) assays were carried out by using the EZ-Zyme chromatin prep kit (Merck Millipore, Billerica, MA) based on the recommended protocols. Anti-*BP1* antibody (NB100-481) (Novus Biologicals, Littleton, CO) and normal immunoglobulin G (IgG) were used for immunoprecipitation. Eluted DNA fragments were analyzed by high-throughput sequencing (ChIP-Seq) performed at Genesky Biotechnologies Inc. (Shanghai, China). All reads were paralleled to the National Center for Biotechnology Information (NCBI) human reference genome build 37 (GRCh37/assembly hg19) using Bowtie2 (http://bowtie-bio.sourceforge.net/bowtie2/index.shtml). The HOMER and MEME software packages were used to conduct a search of consensus binding motifs of *BP1*.

### ATAC-Seq

Assay for transposase-accessible chromatin (ATAC) assay with high-throughput sequencing (ATAC-Seq) allows high-throughput sequencing of open chromatin regions with the help of transposases, which is similar to ChIP-Seq. The tested cells were processed to extract the nucleus, followed by transposition reaction. Separated and purified DNA was analyzed by ATAC-Seq performed at Genesky Biotechnologies Inc. (Shanghai, China). All reads were aligned to the NCBI human reference genome build 37 (GRCh37/assembly hg19) using Bowtie2 (http://bowtie-bio.sourceforge.net/bowtie2/index.shtml). The HOMER and MEME software packages were applied to conduct a search of consensus binding motifs of *DLX7*.

### FISH

Fluorescence in situ hybridization (FISH) assays were applied to determine the location of lncRNAs. Briefly, the exponential phase of cells seeded on slides were cleaned with phosphate-buffered saline (PBS) and fixed in 4% paraformaldehyde. Cells were then treated with the Cy3-labeled *SGMS1-AS1* probe and incubated at 37 °C overnight. Moreover, Cy3-labeled *U6* and *18S* were also included as controls. The probe sequences are listed in Additional file 1: Table S1. After washing with hybridization solution and PBS for 5–10 min, cells were counterstained with 4′,6-diamidino-2-phenylindole (DAPI). Photos were captured by using an Olympus confocal laser scanning microscope.

### Luciferase reporter assay

The putative binding area of human *SGMS1-AS1* and the 3′-untranslated region (UTR) of human *SRPK2* together with the matched mutant fragment were introduced individually into a GV268 vector (Genechem, Shanghai, China). Moreover, *miR-181d-5p* together with the matched mutant fragment was individually inserted into a GV272 vector (Genechem, Shanghai, China). To perform the reporter experiments, the constructed GV268 plasmid (mRNA-3′UTR or LncRNA-binding area) and constructed GV272 plasmid (miRNA) together with *Renilla* luciferase reporter plasmid were cotransfected into 293T cells by using X-tremeGENE HP transfection reagent (Roche, Basel, Switzerland). After 48 h of transfection, the luciferase activities and the firefly luciferase vitalities were examined under the Dual-Luciferase reporter assay system (Promega, Madison, WI). Relative firefly luciferase activity was normalized to *Renilla* luciferase activity to obtain the transfection efficiency.

### RIP

RNA immunoprecipitation (RIP) assays were performed using a Magna RNA-binding protein immunoprecipitation kit (Millipore, Bedford, MA) on the basis of the recommended instructions. Briefly, 2 × 10^7^ K562 cell lysates were incubated with RIP buffer containing magnetic beads conjugated with mouse IgG (negative controls) or human anti-Ago2 antibody (ab32381; Abcam, Cambridge, MA). The specimens were then incubated with proteinase K to isolate the immunoprecipitated RNA. RNAs were extracted and purified, then used for reverse transcription. Finally, RT-qPCR was carried out to confirm the presence of the binding targets. The primers given in Additional file 1: Table S1 were used to detect the *SGMS1-AS1* and *miR-181d-5p* level.

### Bioinformatics analysis

LncLocator (http://www.csbio.sjtu.edu.cn/bioinf/lncLocator/) was applied to predict the location of lncRNA *SGMS1-AS1*. The lncRNA–miRNA interaction was predicted by using starBase (http://starbase.sysu.edu.cn/) and LncBase v.2 (http://carolina.imis.athena-innovation.gr/diana_tools/web/index.php?r=lncbasev2%2Findex). Moreover, TargetScan7.2 (http://www.targetscan.org/vert_72/), miRDB (http://mirdb.org/miRDB/), mirDIP (http://ophid.utoronto.ca/mirDIP/index.jsp), and starBase (http://starbase.sysu.edu.cn/) were utilized to evaluate the miRNA–mRNA interactions.

### Statistics

SPSS 22.0 and GraphPad Prism 5.0 were utilized for statistical analysis. Comparison of continuous variables between two groups was conducted by Independent *T*/Mann–Whitney *U*-tests, whereas comparison of categorical variables was performed by Pearson chi-squared/Fisher exact tests. The receiver operating characteristic (ROC) curve and the area under the ROC curve (AUC) were applied to define the capability of *BP1* and *DLX7* expression for distinguishing CML patients from normal controls. In all analyses, (two-tailed) *P* values less than 0.05 were defined as statistically significant.

## Results

### DNA methylation-mediated differential expression of *DLX4* isoforms in CML

**Fig. 1:**
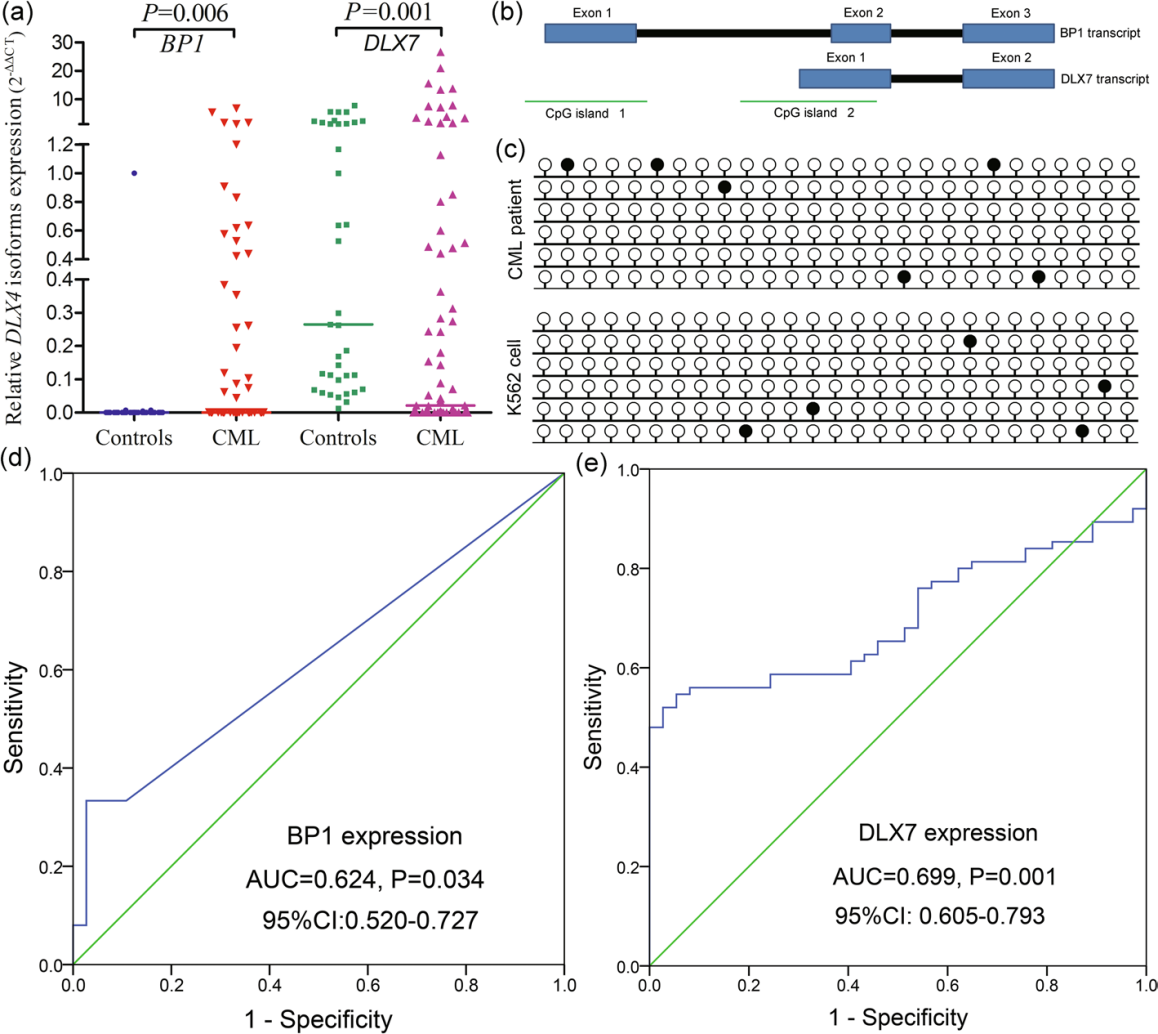
DNA methylation-mediated differential expression of *DLX4* isoforms together with their clinical significance in CML. **a** Relative expression of *DLX4* isoforms *BP1* and *DLX7* in CML patients. *BP1* expression was significantly increased in CML patients, whereas *DLX7* expression was significantly decreased as detected by real-time quantitative PCR. Relative *BP1*/*DLX7* expression values were calculated using the equation 2^∆CT [control−sample (*BP1*/*DLX7*)]^ ÷ 2^∆CT [control−sample (*ABL1*)]^. ∆CT reflects the disparity in CT value between control and target or reference sequences. The bone marrow sample from one normal control that possessed the minimal ∆CT between *BP1*/*DLX7* and *ABL1* transcript was selected as control and defined as 100% expression for *BP1*/*DLX7* transcript. The median level of *BP1*/*DLX7* expression in each group is shown by a horizontal line. **b** The coordinates of CpG islands in *DLX4* gene. **c** Methylation density of *BP1* promoter CpG island (CpG island 1) in a representative CML patient and K562 cell. The CpG island located at the promoter region of *BP1* (CpG island 1) was almost unmethylated in a representative CML patient and K562 cell as detected by bisulfite sequencing. A white circle indicates unmethylated CpG dinucleotide, whereas a black circle indicates methylated CpG dinucleotide. Each line represents an independent clone-sequencing result of BSP product of K562 cell or CML patient. **d** The discriminating value of *BP1* in CML patients. *BP1* expression may serve as a potential biomarker for distinguishing CML patients from controls with an AUC value of 0.624. **e** The discriminating value of *DLX7* in CML patients. *DLX7* expression may serve as a potential biomarker for distinguishing CML patients from controls with an AUC value of 0.699

Following our previous study [27], we determined the expression pattern of *DLX4* isoforms in CML patients. Interestingly, *BP1* expression was significantly increased in CML patients (*P* = 0.006), whereas *DLX7* expression was markedly decreased (*P* = 0.001) (Fig. 1a). To support these results, we further investigated the promoter methylation pattern of *DLX4* isoforms in CML. Notably, independent CpG islands were identified in the promoter region of each *DLX4* isoform (Fig. 1b). As reported in our previous study, the CpG island located at the promoter region of *DLX7* (CpG island 2 in Fig. 1b) was methylated in CML patients and K562 cell line, and was associated with *DLX7* expression [26]. However, the CpG island methylation located at the promoter region of *BP1* (CpG island 1 in Fig. 1b) was undetectable in CML patients and K562 cell line by RT-qMSP, demonstrating that the CpG island located at the promoter region of *BP1* was almost unmethylated in CML patients and K562 cell line. Moreover, the CpG island methylation located at the promoter region of *BP1* was further validated by BSP; the results for K562 cell line and a representative CML patient are shown in Fig. 1c.

To investigate the clinical significance of *DLX4* isoforms in CML, we first performed ROC curve analysis to evaluate the ability of *DLX4* expression to discriminate AML from controls. The AUC value for *BP1* and *DLX7* were 0.624 (95% CI 0.520–0.727, *P* = 0.034, Fig. 1d) and 0.699 (95% CI 0.605–0.793, *P* = 0.001, Fig. 1e), suggesting that they may serve as potential biomarkers for distinguishing CML patients from controls. Based on ROC analysis, *BP1* expression of 0.0256 and *DLX7* expression of 0.0266 were set as cutoff points because the “sensitivity + specificity – 1” reached the highest value. At the cutoff value, the sensitivity and specificity for *BP1* expression were 33.3% and 97.3%, whereas they were 52% and 97.3% for *DLX7* expression. According to the cutoff points, we divided the CML cases into two groups to analyze the clinical significance of both *BP1* and *DLX7* expression. No remarkable differences were found between groups with low and high *BP1* expression among all clinical parameters (*P* > 0.05, Table 1). However, significant differences were observed between the groups with low and high *DLX7* expression in the distribution of clinical stages (*P* = 0.008, Table 1), with low *DLX7* expression appearing with the highest frequency in the blastic crisis (BC) stage [88.9% (8/9)], lower frequency in the accelerated phase (AP) stage [83.3% (5/6)], and the lowest frequency in the chronic phase (CP) stage [43.3% (26/60)]. The incidence of low *DLX7* expression in BC/AP stages was significantly higher than in CP stage [86.7% (13/15) versus 43.3% (26/60), *P* = 0.003]. Moreover, CML patients with low *DLX7* expression exhibited higher *BCR-ABL1* transcript than those with high *DLX7* expression (Table 1).

### *DLX4* isoforms *BP1* and *DLX7* showed opposite function in cell division

**Fig. 2:**
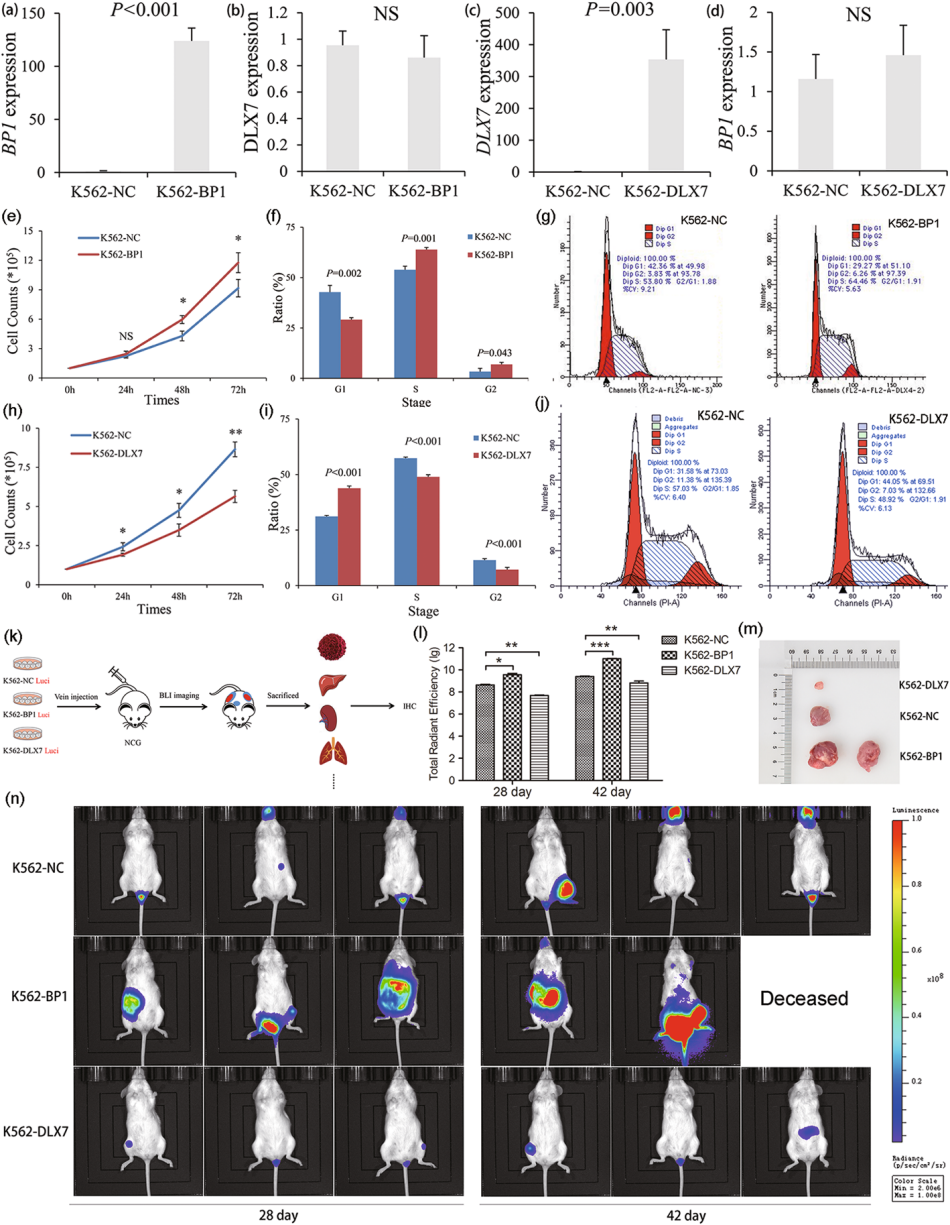
*DLX4* isoforms *BP1* and *DLX7* showed opposite functions in leukemogenesis both in vivo and in vitro. **a** *BP1* expression in K562 cells before and after *BP1* transfection. *BP1* expression was significantly increased after *BP1* transfection in K562 cells as detected by real-time quantitative PCR (RT-qPCR). K562-NC (control) was defined as 100% expression for *BP1* transcript. **b** *DLX7* expression in K562 cells before and after *BP1* transfection. *DLX7* expression was not changed after *BP1* transfection in K562 cells as detected by RT-qPCR. K562-NC (control) was defined as 100% expression for *DLX7* transcript. **c** *DLX7* expression in K562 cells before and after *DLX7* transfection. *DLX7* expression was significantly increased after *DLX7* transfection in K562 cells as detected by RT-qPCR. K562-NC (control) was defined as 100% expression for *DLX7* transcript. **d** *BP1* expression in K562 cells before and after *DLX7* transfection. *BP1* expression was not changed after *DLX7* transfection in K562 cells as detected by RT-qPCR. K562-NC (control) was defined as 100% expression for *BP1* transcript. **e** The proliferation ability in K562 affected by *BP1* overexpression. K562 cells with *BP1* overexpression (K562-BP1) showed a significantly increased proliferation rate than those without *BP1* overexpression (K562-NC). **f** The cell cycle in K562 affected by *BP1* overexpression. K562-BP1 cells showed a significantly higher percentage of S/G2 phase than K562-NC cells. **g** Representative cell cycle diagram of K562-NC and K562-BP1 cells by flow cytometry. **h** The proliferation ability in K562 affected by *DLX7* overexpression. K562 cells with *DLX7* overexpression (K562-DLX7) showed a markedly reduced proliferation rate than those without *DLX7* overexpression (K562-NC). **i** Cell cycle in K562 affected by *DLX7* overexpression. K562-DLX7 cells showed significantly lower percentage of S/G2 phase than K562-NC cells. **j** Representative cell cycle diagram of K562-NC and K562-DLX7 cells by flow cytometry. **k** The flow chart of the in vivo experiment. **l** The tumor load in NCG mice affected by K562 cells with *BP1* and *DLX7* overexpression. The tumor load of K562-BP1 group mice was significantly higher, whereas the tumor load of K562-DLX7 group mice was significantly lower compared with the tumor load of K562-NC group mice via bioluminescence imaging at the 28th day and 42th day. **m** The representative tumor load diagram of NCG mice with K562-NC, K562-BP1, and K562-DLX7 cells injection as detected by bioluminescence imaging. **n** The representative tumor volume of NCG mice with injection of K562-NC, K562-BP1, and K562-DLX7 cells. *P < 0.05; **P < 0.01; ***P < 0.001; NS, no significance

Since the *DLX4* isoforms *BP1* and *DLX7* showed a differentially expressed pattern in CML, we next investigated the potential role of these two *DLX4* isoforms in cell division. Because it is hard to knockdown *BP1* and *DLX7* expression alone by RNA interference, we performed gain-of-function experiments in K562 cells with *BP1*/*DLX7* overexpression by infection with lentivirus (Genechem, Shanghai, China) carrying the complete CDS of *BP1* and *DLX7*. The establishment of K562 cells stably overexpressing *BP1* was proved by both RT-qPCR (Fig. 2a, b) and western blot (Additional file 12), whereas K562 cells stably overexpressing *DLX7* were only confirmed by RT-qPCR because of the lack of specific antibodies (Fig. 2c, d). As expected, overexpression of *BP1* significantly increased the proliferation rate of K562 cells together with S/G2 promotion (Fig. 2f–h), whereas *DLX7* overexpression markedly decreased the proliferation rate of K562 cells together with G1 arrest (Fig. 2i–k). Collectively, the functional experiments in vitro demonstrated the promitotic effects of *BP1* but the antimitotic effects of *DLX7* in leukemogenesis.

### Functional role of *DLX4* isoforms *BP1* and *DLX7* in a mouse xenograft model

To further determine the role of *DLX4* isoforms *BP1* and *DLX7 *in vivo, we constructed xenograft mouse models by tail vein injection of K562-NC/K562-BP1/K562-DLX7 cells in NCG mice. The experimental procedures are shown in Fig. 2m. Firstly, the progression of the tumor load in mice was tracked using bioluminescence imaging, revealing that the tumor load of the K562-BP1 group mice was higher, whereas the tumor load of K562-DLX7 group mice was lower as compared with the tumor load of K562-NC group mice (Fig. 2n, o). Moreover, the tumor size in K562-BP1/K562-DLX7/K562-NC group mice is shown in Fig. 2p. Overall, the functional studies in vivo further support the tumor-promoting role of *BP1* but the tumor-suppressing role of *DLX7* in leukemogenesis.

### Molecular mechanism of *DLX4* isoforms *BP1* and *DLX7* in leukemogenesis

**Fig. 3:**
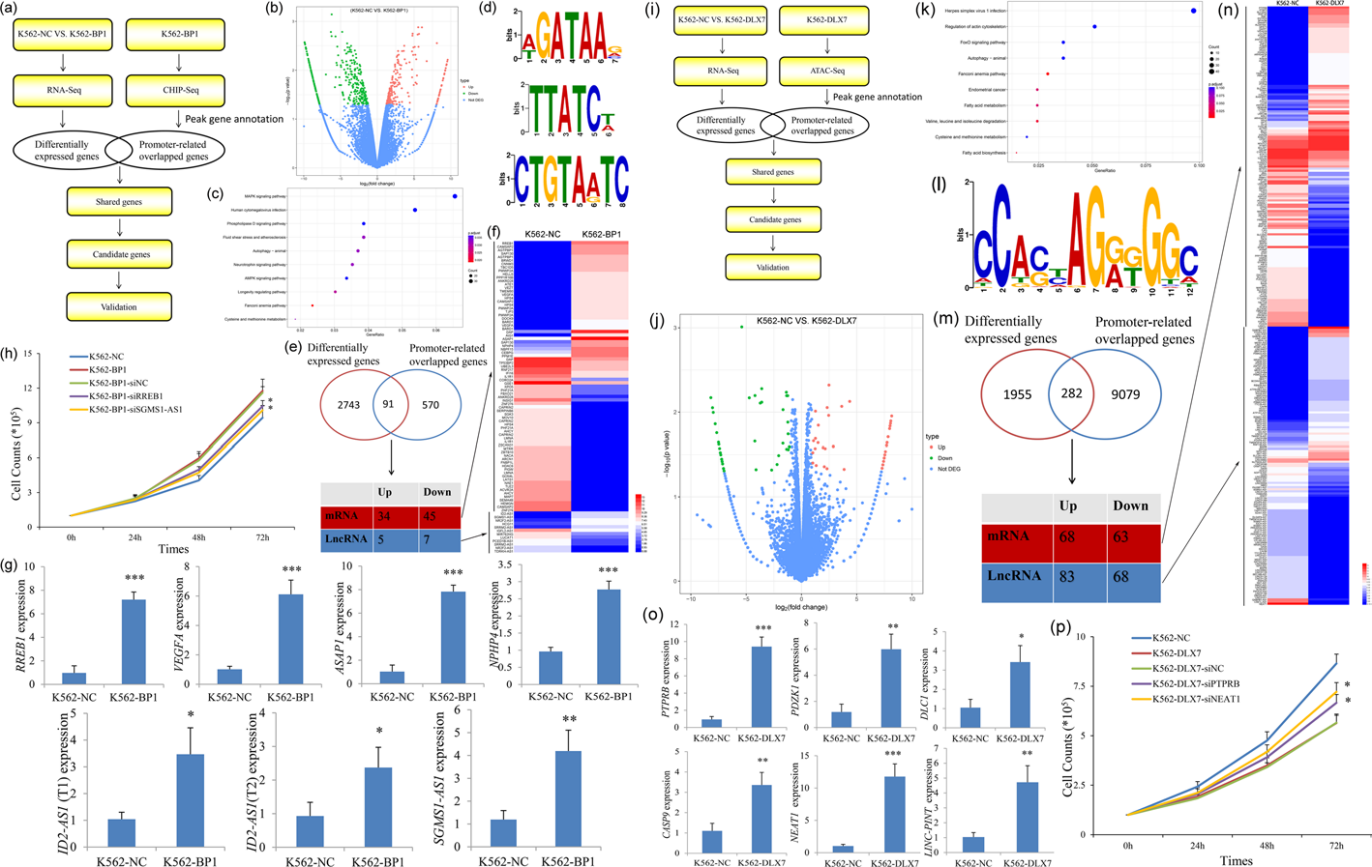
Molecular mechanism of *DLX4* isoforms *BP1* and *DLX7* in leukemogenesis. **a** The flow chart of the molecular mechanism experiment. **b** Volcano plot of DEGs between K562-BP1 and K562-NC cells. **c** KEGG analysis of differentially expressed genes between K562-BP1 and K562-NC cells. **d** The top three motifs analyzed with K562-BP1 cells by ChIP-Seq. **e**, **f** Potential downstream targets by Venn analysis of ChIP-Seq and RNA-Seq. **g** Validation of the selected gene expression by RT-qPCR. **h** The proliferation ability of K562-BP1 cells affected by *BP1* downstream target gene knockdown. Both K562-BP1-siRREB1 and K562-BP1-siSGMS1-AS1 cells showed significantly reduced proliferation rate compared with K562-BP1-siNC cells. **i** Flow chart of the molecular mechanism experiment. **j** Volcano plot of DEGs between K562-DLX7 and K562-NC cells. **k** KEGG analysis of DEGs between K562-DLX7 and K562-NC cells. **l** The top one motif analyzed with K562-DLX7 cells by ATAC-Seq. **m**, **n** Potential downstream targets by Venn analysis of ATAC-Seq and RNA-Seq. **o** Validation of selected gene expression by RT-qPCR. **p** Proliferation ability of K562-DLX7 cells affected by *DLX7* downstream target gene knockdown. Both K562-DLX7-siPTPRB and K562-DLX7-siNEAT1 cells showed markedly increased proliferation rate compared with K562-DLX7-siNC cells. *P < 0.05; **P < 0.01; ***P < 0.001

Firstly, to explore how the transcription factor *BP1* exerts its function, we performed ChIP-Seq and RNA-Seq to identify candidate downstream genes in leukemogenesis (Fig. 3a). Using RNA-Seq, a total of 2834 genes were identified to be differentially expressed after *BP1* overexpression (Fig. 3b and Additional file 2: Table S2, Additional file 3: Table S3 and Additional file 4: Table S4). Kyoto Encyclopedia of Genes and Genomes (KEGG) analysis revealed that the differentially expressed genes (DEGs) were significantly enriched in several cancer-related signaling pathways such as MAPK and AMPK signaling (Fig. 3c). Moreover, ChIP-Seq results revealed that the transcription factor *BP1* may bind to the promoter region of 661 genes (Additional file 5: Table S5). Motif analysis identified the top three motifs in BP1-bound promoters, which is shown visually in Fig. 3d. Venn analysis of the RNA-Seq and ChIP-Seq results showed that a total of 91 genes including 79 mRNA and 12 lncRNAs may act as direct downstream targets of *BP1* (Fig. 3e, f). Four mRNAs (*RREB1*, *VEGFA*, *ASAP1*, and *NPHP4*) as well as three lncRNAs (*ID2-AS1 transcript 1*, *ID2-AS1 transcript 2*, and *SGMS1-AS1*) were further validated by RT-qPCR (Fig. 3g). To further confirm the biological association of *RREB1* and *SGMS1-AS1* with *BP1*, we next performed rescue experiments during luekemogenesis caused by *BP1* overexpression. As expected, knockdown of *RREB1* and *SGMS1-AS1* expression by siRNA markedly impaired the proliferation after *BP1* overexpression in K562 cells (Fig. 3h).

Secondly, because of a lack of specific antibodies, we used ATAC-Seq and RNA-Seq to identify candidate downstream genes in leukemogenesis (Fig. 3i). RNA-Seq revealed a total of 2277 genes identified as differentially expressed after *DLX7* overexpression (Fig. 3j and Additional file 6: Table S6, Additional file 7: Table S7, Additional file 8: Table S8). KEGG analysis revealed that the DEGs were significantly enriched in several cancer-related signaling pathways such as FoxO signaling (Fig. 3k). Additionally, *DLX7* may bind to the promoter region of 9361 genes by ATAC-Seq (Additional file 9: Table S9). Motif analysis identified the top one motif in DLX7-bound promoters, as shown visually in Fig. 3l. Venn analysis of the RNA-Seq and ATAC-Seq results showed that a total of 282 genes including 151 mRNA and 131 lncRNAs may act as direct downstream targets of *DLX7* (Fig. 3m, n). Four mRNAs (*PTPRB*, *PDZK1*, *DLC1*, and *CASP9*) as well as two lncRNAs (*NEAT1* and *LINC-PINT*) were further validated by RT-qCR (Fig. 3o). To further confirm the biological connections of *PTPRB* and *NEAT1* with *DLX7*, we next performed rescue experiments during luekemogenesis caused by *DLX7* overexpression. As expected, knockdown of *PTPRB* and *NEAT1* expression by siRNA partially revived the proliferation after *DLX7* overexpression in K562 cells (Fig. 3p).

### Identification of an lncRNA–miRNA–mRNA network in leukemogenesis caused by *BP1* overexpression

**Fig. 4:**
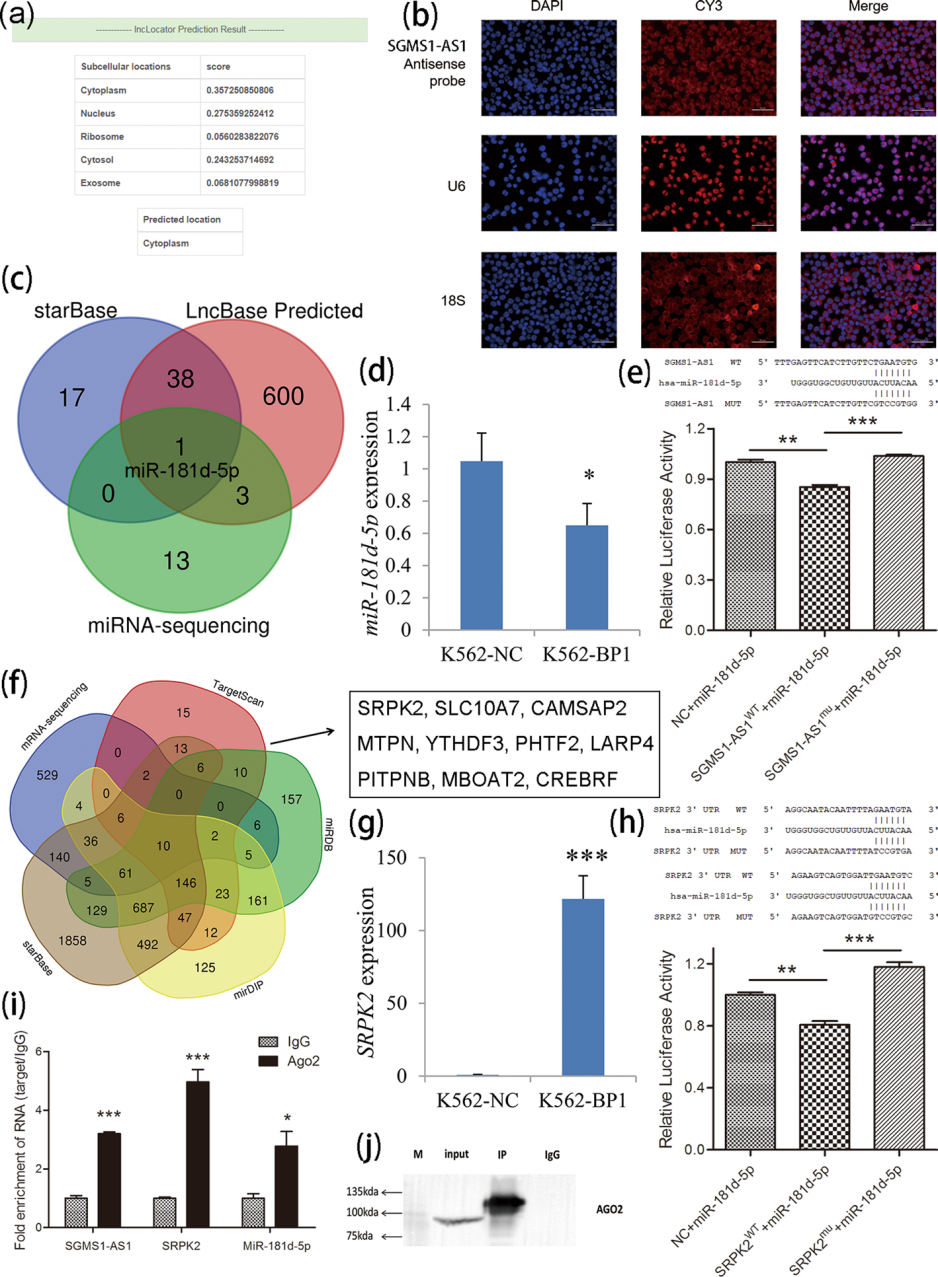
Identification of the *SGMS1-AS1*/*miR-181d-5p*/*SRPK2* ceRNA network activated by *BP1* overexpression. **a** Prediction of the location of *SGMS1-AS1* by IncLocator (http://www.csbio.sjtu.edu.cn/bioinf/lncLocator). **b** Validation of the location of *SGMS1-AS1* by RNA FISH. *SGMS1-AS1* was localized mainly in the cytoplasm of K562 cells. **c** Prediction of the downstream miRNA of *SGMS1-AS1* by Venn analysis of LncBase Predicted (http://carolina.imis.athena-innovation.gr/diana_tools/web/index.php?r=lncbasev2%2Findex), starBase (http://starbase.sysu.edu.cn/), and miRNA-Seq. **d** Validation of the selected gene expression by RT-qPCR. **e** Dual luciferase experiment of *SGMS1-AS1* binding to *miR-181d-5p*. Overexpression of *miR-181d-5p* significantly reduced the luciferase activity of the wild-type *SGMS1-AS1* vector but not of the mutated *SGMS1-AS1* vector. **f** Prediction of the downstream mRNA of *SGMS1-AS1* by Venn analysis of mRNA-Seq, miRDB (http://mirdb.org/miRDB/), TargetScan (http://www.targetscan.org/vert_72/), mirDIP (http://ophid.utoronto.ca/mirDIP/index.jsp), and starBase (http://starbase.sysu.edu.cn/). **g** Validation of the selected gene expression by RT-qPCR. **h** Dual luciferase experiment of *miR-181d-5p* binding to *SRPK2*. Overexpression of *miR-181d-5p* markedly reduced the luciferase activity of the wild-type 3′-UTR of *SRPK2* vector but not of the mutated 3′-UTR of *SRPK2* vector. **i** The *SGMS1-AS1*/*miR-181d-5p*/*SRPK2* expression detected by RT-qPCR after Ago2-RIP. Endogenous *SGMS1-AS1*, *miR-181d-5p*, and *SRPK2* was preferentially enriched in Ago2-RIPs compared with control IgG-RIPs. **j** RNA immunoprecipitation (RIP) efficiency confirmation detected by western blot. *P < 0.05; **P < 0.01; ***P < 0.001

Given the results above, most of the differentially expressed mRNAs together with miRNAs that were not identified as direct downstream targets of *BP1* greatly aroused our attention. Based on the fact that several lncRNAs were verified as downstream targets of *BP1*, we deduced that the miRNAs and mRNAs were downstream targets of the activated lncRNAs and may be indirectly activated by *BP1*. It is well known that lncRNAs located in cytoplasm may work through a competing endogenous RNA (ceRNA) (lncRNA–miRNA–mRNA) network. Herein, we verified that an lncRNA *SGMS1-AS1* directly activated by *BP1* was anticipated to be located mainly in the cytoplasm of all available cell types by using lncLocator (http://www.csbio.sjtu.edu.cn/bioinf/lncLocator/) (Fig. 4a). Further LncRNA-FISH assays confirmed that *SGMS1-AS1* was localized mainly in the cytoplasm of K562 cells (Fig. 4b). To explore the downstream miRNAs, we identified miRNAs that can bind with *SGMS1-AS1* by using publicly available online tools (LncBase Predicted v.2 and starBase) together with miRNA sequencing. The results revealed that only *miR-181d-5p* was shared in all three conditions (Fig. 4c and Additional file 10: Table S10). Additionally, the reduced expression of *miR-181d-5p* after *BP1* overexpression in K562 cells was further validated by RT-qPCR (Fig. 4d). Moreover, the dual-luciferase reporter assays revealed that overexpression of *miR-181d-5p* significantly reduced the luciferase activity of the wild-type *SGMS1-AS1* vector but not of the mutated *SGMS1-AS1* vector (Fig. 4e). Together, these findings prove a direct association between *SGMS1-AS1* and *miR-181d-5p* in leukemogenesis.

To explore the downstream targets of *SGMS1-AS1*/*miR-181d-5p*, we predicted mRNAs that can bind with *miR-181d-5p* by using publicly available online tools (miRDB, TargetScan 7.2, starBase, and mirDIP) together with RNA sequencing. We screened a total of ten mRNAs (*SLC10A7*, *CAMSAP2*, *MTPN*, *YTHDF3*, *PHTF2*, *PITPNB*, *MBOAT2*, *LARP4*, *SRPK2*, and *CREBRF*) that may act as direct targets of *SGMS-AS1*/*miR-181d-5p* (Fig. 4f and Additional file 11: Table S11). A significant cancer-associated gene *SRPK2* was selected for further study. We first validated the increased expression of *SRPK2* after *BP1* overexpression in K562 cells by RT-qPCR (Fig. 4g). Next, by dual-luciferase reporter assays, overexpression of *miR-181d-5p* markedly decreased the luciferase activity of the wild-type 3′-UTR of *SRPK2* vector but not of the mutated 3′-UTR of *SRPK2* vector (Fig. 4h). These results suggest that *SGMS1-AS1* may “absorb” *miR-181d-5p* to regulate *SRPK2* and thus be involved in leukemogenesis.

Because Ago2 is a core component of the RNA-induced silencing complex (RISC) involved in miRNA-mediating mRNA destabilization or translational repression, we performed RIP assays by using an anti-Ago2 antibody, which demonstrated that endogenous *SGMS1-AS1* together with *miR-181d-5p* and *SRPK2* was preferentially enriched in Ago2-RIPs compared with control IgG-RIPs (Fig. 4i, j). All these findings suggest that *SGMS1-AS1* functions via ceRNA network to play its biologic role during leukemogenesis.

**Fig. 5:**
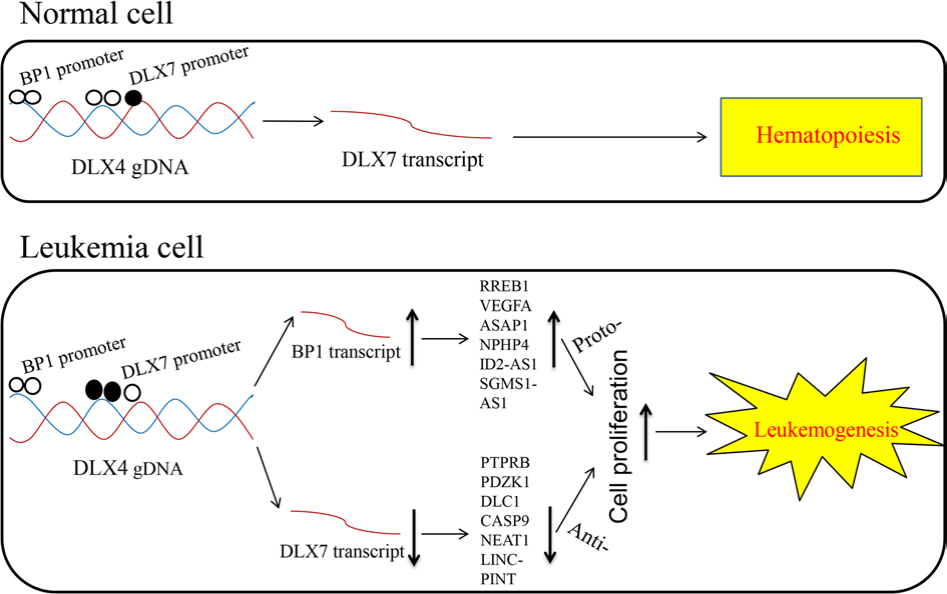
Schematic diagram of *DLX4* gene isoforms in promoting leukemogenesis

Combining these findings, a schematic diagram of the roles of *DLX4* gene isoforms in promoting leukemogenesis is presented in Fig. 5.

## Discussion

The expression pattern of *DLX4* and its clinical significance have been demonstrated in several human cancers. Gao et al. revealed that *DLX4* expression was dramatically increased in patients with hepatocellular carcinoma and was associated with poor prognosis [16]. Man et al. demonstrated that overexpression of *BP1* independently affected disease-free survival in patients with NSCLC [17]. Moreover, mounting evidence has shown the adverse effect of *DLX4*/*BP1* overexpression on clinical outcome in patients with breast cancer [12–15]. Importantly, Haga et al. also reported the overexpression pattern of *BP1* in all types of acute leukemia [18]. However, several studies have also shown the hypermethylation of *DLX4* with a role of transcriptional inactivation in stage I NSCLC, uterine cervical LSILs, breast cancer, and CLL [19–23]. Moreover, *DLX4* hypermethylation was found to be associated with disease progression in uterine cervical LSILs and NSCLC [22, 23]. Taking these results together, the expression pattern of *DLX4* seems to be “contradictory” when contrasted with the methylation condition of *DLX4* in human cancers, especially breast cancer. In-depth analysis in previous studies regarding *DLX4* methylation has focused on the CpG islands located in the promoter region of *DLX7* but not the promoter region of *BP1* [19–23]. The methylation pattern of the CpG island located at the promoter region of *BP1* has rarely been studied. Our preliminary studies also confirmed the phenomenon of *DLX4* hypermethylation in MDS, AML, and CML [24–26]. Moreover, *DLX4* methylation with its role in silencing *DLX7* expression but not *BP1* expression was further verified in AML and CML [25, 26]. In addition, *BP1* and *DLX7* showed opposite expression patterns in AML patients; that is, *BP1* expression was increased, whereas *DLX7* expression was decreased [27]. In the work described herein, we further detected the expression pattern of *BP1* and *DLX7* in CML patients, with results similar to AML patients [27]. Moreover, the CpG islands located at the promoter region of *BP1* were nearly unmethylated in CML. These results further support that the DNA methylation-mediated differential expression pattern of *DLX4* isoforms may play a different role in leukemogenesis.

Regarding the role of *DLX4* in tumorigenesis, most previous studies have mainly focused on *DLX4* isoform 1, and thus *BP1* is also called *DLX4* in some studies. Zhang et al. demonstrated that *DLX4* activated expression of *TWIST* to promote epithelial-to-mesenchymal transition (EMT), cancer migration, invasion, and metastasis in breast cancer [33]. Alternatively, *BP1* overexpression markedly enhanced cell proliferation and metastatic potential in estrogen receptor (ER)-negative Hs578T breast cancer cells [34]. Haria et al. showed that *DLX4* induced CD44 expression by stimulating interleukin (IL)-1β-mediated nuclear factor kappa-light-chain-enhancer of activated B cells (NF-κB) activity, thereby promoting peritoneal metastasis of ovarian cancer [35]. Moreover, *DLX4* played an oncogenic role in clear cell renal cell carcinoma via inducing proliferation and EMT, and was associated with poor prognosis [36]. *DLX4* isoform *BP1* overexpression promoted cell proliferation, migration in endometrial cancer with clinically prognostic effect [37]. However, the direct role of *DLX7* was poorly determined. In the current work, we systemically investigated the direct role of *BP1* and *DLX7* during leukemogenesis by in vivo and in vitro studies. Our study demonstrated that *DLX4* isoforms *BP1* and *DLX7* have opposite functions in leukemogenesis, with *BP1* playing an oncogenic role whereas *DLX7* plays a tumor suppressive role in leukemogenesis. Interestingly, *DLX4* isoforms *BP1* and *DLX7* have distinct functions in the regulation of the b-globin gene [38].

The potential mechanism of *DLX4*, as a transcription factor, in carcinogenesis has been investigated. Most previous studies have explored the potential downstream targets of *DLX4* by single-gene identification based on ChIP-PCR. For instance, Kluk et al. disclosed that *BP1* homeoprotein repressed *BRCA1* expression by direct binding to its first intron in sporadic breast cancer [39]. Alternatively, *BP1* transcriptionally activates bcl-2 and inhibits tumor necrosis factor (TNF)-α-induced cell death in MCF7 breast cancer cells [40]. Trinh et al. showed that *DLX4* blocked the antiproliferative effect of transforming growth factor (TGF)-β by inhibiting TGF-β-mediated induction of p15^Ink4B^ and p21^WAF1/Cip1^ expression and inducing expression of c-myc independently of TGF-β/Smad signaling [41]. Moreover, *DLX4* promoted nasopharyngeal carcinoma progression via inducing *YB-1* expression [42]. In this study, we used ChIP-Seq/ATAC-Seq together with RNA-Seq to identify the underlying targets of *DLX4* in leukemogenesis by whole-genome-scale screening. Interestingly, although the two isoforms *BP1* and *DLX7* are predicted to share a common homeodomain, the binding motifs were discrepant for *BP1* and *DLX7*. A possible mechanism is that the locus occupancy is mediated by other proteins that bind DNA directly. Berger et al. revealed that variation in homeodomain DNA binding sequence recognition may be a factor in functional diversity and evolutionary success [43]. Similarly, Song et al. using ChIP on chip (ChIP-on-chip) and gene expression microarray assays to screen the downstream targets of *BP1* in ER– breast cancer cells [44]. A total of 18 genes were identified and verified, of which some were involved in a variety of tumorigenic pathways in breast cancer development and progression [44]. Although *VEGFA* was identified as a downstream target of *DLX4* in the studies by Song et al. [44] and Hara et al. [45] as well as our study, most of the genes were not identified by our investigation. These results suggest that *BP1* could regulate different downstream target among different types of human cancer.

On the basis of whole-genome study, most of the differentially expressed mRNAs together with miRNAs that were not identified as direct downstream targets of *BP1* greatly aroused our attention. An emerging phenomenon is that one of the molecular mechanisms by which lncRNAs regulate gene expression is to interact with miRNA as ceRNAs that bind to miRNA response elements and protect miRNAs from binding to and repressing target mRNAs [46–48]. Accordingly, we deduced that the miRNAs and mRNAs were downstream targets of the activated lncRNAs and may be indirectly activated by *BP1*. In this study, we identified a *BP1*/*SGMS1-AS1*/*miR-181d-5p*/*SRPK2* ceRNA network in leukemogenesis. Although there are no studies regarding *SGMS1-AS1* in leukemogenesis, several studies have indicated the role of *miR-181d* and *SRPK2* in AML biology. Su et al. reported the direct role of the *miR-181* family in normal hematopoiesis and AML development [49]. Moreover, *miR-181d*/RBP2/NF-κB p65 feedback regulation promoted CML progression into blast crisis [50]. Additionally, Jang et al. revealed that *SRPK2* promoted leukemia cell proliferation by phosphorylating acinus and regulating cyclin A1 [51]. Collectively, all these results suggest the crucial role of the *BP1*/*SGMS1-AS1*/*miR-181d-5p*/*SRPK2* network in leukemogenesis.

## Conclusion

Taken together, our findings reveal that DNA methylation-mediated differential expression of *DLX4* isoforms *BP1* and *DLX7* have opposite functions in leukemogenesis. *BP1* plays an oncogenic role in leukemia development, whereas *DLX7* acts as a tumor suppressor gene. Moreover, the *SGMS1-AS1*/*miR-181d-5p*/*SRPK2* ceRNA network activated by *BP1* plays a significant role in leukemogenesis. These results suggest *DLX4* as a therapeutic target in antileukemia therapy.

## Supplementary Information

**Additional file 1: Table S1.** Primers and sequences used for RT-qPCR, RT-qMSP, BSP, RIP-PCR, LncRNA probe, and RNA interference.

**Additional file 2: Table S2.** mRNA expression analyzed by RNA-seq in K562 cell after *BP1* overexpression (original data).

**Additional file 3: Table S3.** lncRNA expression analyzed by RNA-seq in K562 cell after *BP1* overexpression (original data).

**Additional file 4: Table S4.** miRNA expression analyzed by RNA-seq in K562 cell after *BP1* overexpression (original data).

**Additional file 5: Table S5.** PEAKs and PEAK-annotated genes associated with *BP1* analyzed by ChIP-seq in K562 cell (original data).

**Additional file 6: Table S6.** mRNA expression analyzed by RNA-seq in K562 cell after *DLX7* overexpression (original data).

**Additional file 7: Table S7.** mRNA expression analyzed by RNA-seq in K562 cell after *DLX7* overexpression (original data).

**Additional file 8: Table S8.** mRNA expression analyzed by RNA-seq in K562 cell after *DLX7* overexpression (original data).

**Additional file 9: Table S9.** PEAKs and PEAK-annotated genes associated with *DLX7* analyzed by ATAC-seq in K562 cell (original data).

**Additional file 10: Table S10.** Identification miR-181d-5p as a downstream target of SGMS1-AS1 by Venn analysis of publicly available online tools (LncBase Predicted v.2 and starBase) together with RNA sequencing (miRNA).

**Additional file 11: Table S11.** Identification of downstream targets of miR-181d-5p by Venn analysis of publicly available online tools (miRDB, TargetScan 7.2, starBase, and mirDIP) together with RNA sequencing (mRNA).

**Additional file 12.** Confirmation of BP1 overexpression after BP1 transfection in K562 cells detected by western blot (original figure).

## Data Availability

The datasets used and/or analyzed during the current study are available from the corresponding author on reasonable request.

## Abbreviations

AML: Acute myeloid leukemia
CML: Chronic myeloid leukemia
MDS: Myelodysplastic syndromes
MPN: Myeloproliferative neoplasms
CLL: Chronic lymphocytic leukemia
LSILs: Low-grade squamous intraepithelial lesions
NSCLC: Non-small cell lung cancer
BM: Bone marrow
RT-qPCR: Real-time quantitative PCR
RT-qMSP: Real-time quantitative methylation-specific PCR
BSP: Bisulfite sequencing PCR
CDS: Coding sequences
ChIP: Chromatin immunoprecipitation
ChIP-Seq: Chromatin immunoprecipitation with high-throughput sequencing
ATAC-Seq: Assay for transposase-accessible chromatin with high-throughput sequencing
FISH: Fluorescence in situ hybridization
RIP: RNA immunoprecipitation
ROC: Receiver operating characteristic
AUC: Area under the ROC curve
ceRNA: Competing endogenous RNA
BC: Blastic crisis
AP: Accelerated phase
CP: Chronic phase
KEGG: Kyoto Encyclopedia of Genes and Genomes
DEGs: Differentially expressed genes
RISC: RNA-induced silencing complex
EMT: Epithelial-to-mesenchymal transition
