# Supplementary material for: DNA methylation-mediated differential expression of DLX4 isoforms has opposing roles in leukemogenesis
Source: Cell Mol Biol Lett. 2022 Jul 26;27:59. doi: 10.1186/s11658-022-00358-0 (PMC9327205; doi:10.1186/s11658-022-00358-0)
Supplement: Supplementary file 3 — Additional file 3: Table S3. lncRNA expression analyzed by RNA-seq in K562 cell after BP1 overexpression (original data). [file 11658_2022_358_MOESM3_ESM.docx]

**Supplementary Table S1: Primers and sequences used for RT-qPCR, RT-qMSP, BSP, RIP-PCR, LncRNA probe and RNA interfere.**

| Primer name | Primer sequence (5’to 3’) |
| --- | --- |
| RT-qPCR primers |  |
| *BP1*-F | TACCCGCTTGGCTTGTCC |
| *BP1*-R | AACGCTGGTTTAGGTGCTG |
| *DLX7*-F | CCCCTACACCGTGTTGTGC |
| *DLX7*-R | TTATACTTGGAGCGTTTGTTCTGA |
| *RREB1*-F | CTTCCACGACTTAGGATTCACG |
| *RREB1*-R | CGCCACATGGGTCTGCTT |
| *VEGFA*-F | GAAGGAGGAGGGCAGAAT |
| *VEGFA*-R | CACAGGATGGCTTGAAGAT |
| *ASAP1*-F | GGCAGACAACGATGACGAG |
| *ASAP1*-R | AAGGACACTGGAAAGACCC |
| *NPHP4*-F | CCATCTGCGAGTGTCTTTCTTTG |
| *NPHP4*-R | AGGCTCCCATCCCGTTTC |
| *SGMS1-AS1*-F | AACCTGAACGGTGGTCTTC |
| *SGMS1-AS1*-R | GCAGTGCCCTTCTGATTG |
| *ID2-AS1*(T1)-F | CTGGGGTGGTAAATAGAGTA |
| *ID2-AS1*(T1)-R | TCTGGCTGGACAGGAAAC |
| *ID2-AS1*(T2)-F | AGCCACCAACTGACCAAG |
| *ID2-AS1*(T2)-R | CATCCACCCACTCCTGAC |
| *PTPRB*-F | CAATCGGAACACGACAGACA |
| *PTPRB*-R | TGAGTTACCACCCACAGCAC |
| *PDZK1*-F | AGGGTCTCATTCTGTTGCC |
| *PDZK1*-R | CCTCGGTGTCCTTCTCAA |
| *DLC1*-F | GAATAACGGCTCTGTGAACT |
| *DLC1*-R | TCCGACCACTGATTGACTA |
| *CASP9*-F | TCGCTAATGCTGTTTCGG |
| *CASP9*-R | TCCCTCTTCCTCCACTGTTC |
| *NEAT1*-F | ACATTGACCAACGCTTTATT |
| *NEAT1*-R | GACTCCAACAGCCACTCG |
| *LINC-PINT*-F | CGAGTTAGAAGAGTTGGGATA |
| *LINC-PINT*-R | TGAGATGGTTCCAGTCCC |
| *ABL*-F | TCCTCCAGCTGTTATCTGGAAGA |
| *ABL*-R | TCCAACGAGCGGCTTCAC |
| *miR-181d-5p*-F | AACATTCATTGTTGTCGGTGGGT |
| *miR-181d-5p* -R | Universal primer in miScript SYBR green PCR Kit |
| *U6*-F | GTGCTCGCTTCGGCAGCACATATAC |
| *U6*-R | AAAATATGGAACGCTTCACGAATTTG |
|  |  |
| RT-qMSP primers |  |
| *BP1*-MF | TAGATCGGTTTGGAGTATGC |
| *BP1*-MR | AACGTCCCGAATTAACTACC |
| *BP1*-UF | AGGTAGATTGGTTTGGAGTATGT |
| *BP1*-UR | TTAAACATCCCAAATTAACTACC |
| *ALU*-F | TTAGGTATAGTGGTTTATATTTGTAATTTTAGTA |
| *ALU*-R | ATTAACTAAACTAATCTTAAACTCCTAACCTCA |
|  |  |
| BSP primers |  |
| *BP1*-BF | GGGAGATTGTAAATGTAGATTTTTTG |
| *BP1*-BR | ATACCCCCCATAACTTTCCC |
|  |  |
| RIP-PCR primers |  |
| *SGMS1-AS1*-F | CACCAGGCTTGTTCTGAGGT |
| *SGMS1-AS1*-R | TGGATCTCTCGCTCCATGA |
| *miR-181d-5p*-F | AACATTCATTGTTGTCGGTGGGT |
| *miR-181d-5p* -R | Universal primer in miScript SYBR green PCR Kit |
| *SRPK2*-F | CTTTGCAGGTTGCACACA |
| *SRPK2*-R | CCACTGACTTCTAAAAGAGG |
|  |  |
| LncRNA probe |  |
| *U6* | CY3-CACGAAUUUGCGUGUCAUCCUU |
| *18S* | CY3-AUGCUUUCGCUCUGGUCCGUCUUGC |
| *SGMS1-AS1* | CY3-AGUGCCCUUCUGAUUGUUGAUGGUGGAGUAUG |
|  |  |
| siRNA sequences |  |
| si*RREB1*-F | UUUCUUUGUGUUAUCAAGCUG |
| si*RREB1*-R | GCUUGAUAACACAAAGAAAAC |
| si*SGMS1-AS1*-F | AUACCUUUUGGUUUCCUUGCU |
| si*SGMS1-AS1-R* | CAAGGAAACCAAAAGGUAUAA |
| si*PTPRB*-F | UUCUCAUUUUUGAAAAGACAC |
| si*PTPRB*-R | GUCUUUUCAAAAAUGAGAAAG |
| si*NEAT1*-F | UAGAGAAAAGUCCAAAAGGAG |
| si*NEAT1*-R | CCUUUUGGACUUUUCUCUAGG |
