# Supplementary figures and images for: DNA methylation-mediated differential expression of DLX4 isoforms has opposing roles in leukemogenesis
Source: Cell Mol Biol Lett. 2022 Jul 26;27:59. doi: 10.1186/s11658-022-00358-0 (PMC9327205; doi:10.1186/s11658-022-00358-0)

BP1

Marker

K562-NC

K562-BP1

Tubulin

Marker

K562-NC

K562-BP1

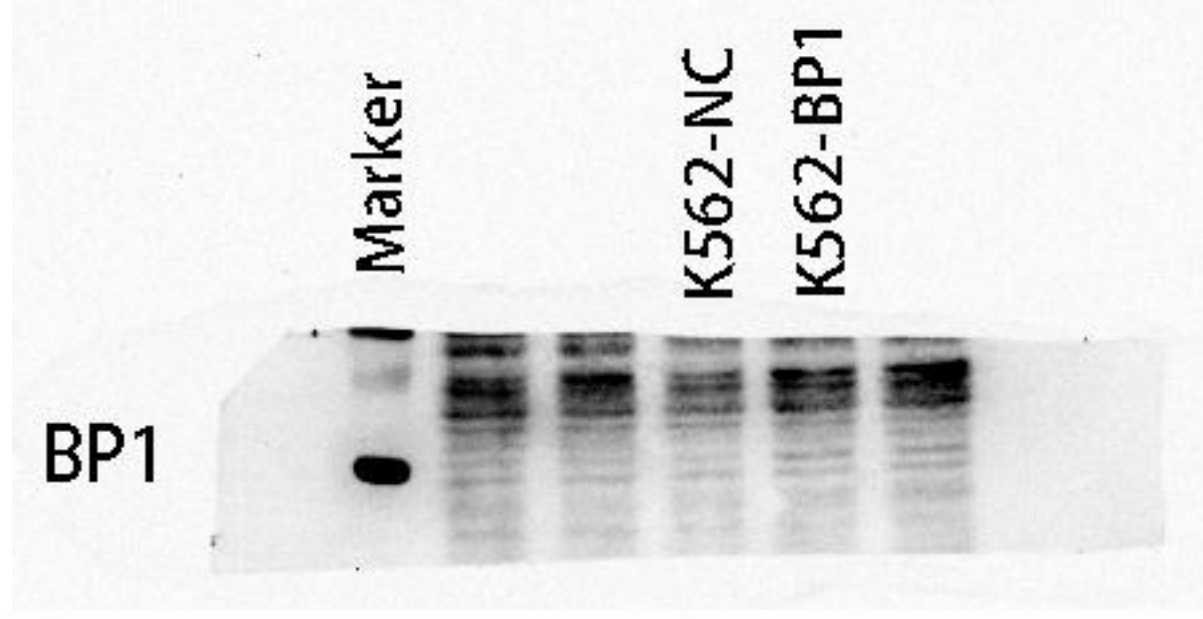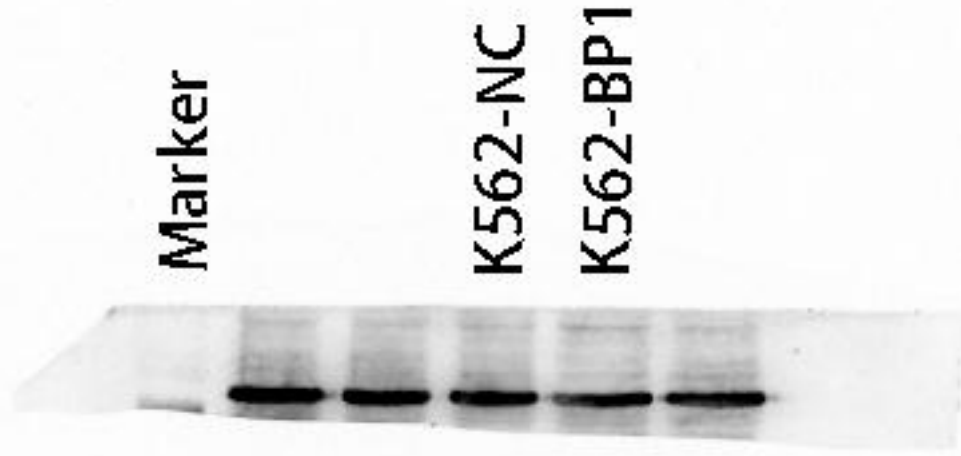

Supplement: Supplementary file 12 — Additional file 12. Confirmation of BP1 overexpression after BP1 transfection in K562 cells detected by western blot (original figure). [file 11658_2022_358_MOESM12_ESM.pdf]
